# Invasive Fungal Granuloma of the Skull Base Mimicking Spindle Cell Neoplasm in a Young Immunocompetent Patient: A Diagnostic Challenge

**DOI:** 10.7759/cureus.92672

**Published:** 2025-09-18

**Authors:** Huzaifa Ali Khan, Qaiser Yaseen, Hafiz Muhammad Usama Amjad, Sajjad Dasti

**Affiliations:** 1 Neurosurgery, Nishtar Medical University Hospital, Multan, PAK; 2 Respiratory Medicine, Aintree University Hospital, Liverpool, GBR; 3 Gastroenterology, Southend University Hospital, Southend, GBR

**Keywords:** biopsy misdiagnosis, fungal granuloma, infectious pseudotumor, skull base mass, spindle cell neoplasm

## Abstract

Skull base lesions presenting with proptosis and sinonasal involvement in young patients are commonly attributed to vascular neoplasms such as juvenile nasopharyngeal angiofibroma. However, rare infectious etiologies such as invasive fungal granulomas may present with overlapping radiological features, leading to diagnostic challenges. We report a rare case of a 19-year-old immunocompetent male presenting with progressive bilateral proptosis and a large skull base mass involving the clivus, nasal cavity, and orbits. Initial imaging suggested a vascular neoplasm, and biopsy revealed a spindle cell lesion with inconclusive immunohistochemistry. Fungal cultures were negative. The patient was lost to follow-up but returned four months later with worsening symptoms. Repeat biopsy demonstrated necrotizing granulomatous inflammation with septate, acute-angle branching fungal hyphae, consistent with *Aspergillus* species on GMS (Grocott methenamine silver) and PAS (periodic acid-Schiff) staining. The final diagnosis was invasive fungal sinusitis mimicking a skull base neoplasm. The patient underwent surgical debridement followed by antifungal therapy with voriconazole and showed clinical improvement. This case underscores the importance of considering fungal granulomas in the differential diagnosis of skull base lesions, even in immunocompetent individuals. It highlights the critical role of repeat biopsy, fungal stains, and a multidisciplinary approach in arriving at an accurate diagnosis when imaging and histology are inconclusive.

## Introduction

The skull base is a complex and critical area at the bottom of the cranium, supporting the brain, and it contains the neurovascular structures entering or exiting the skull. A variety of tumors and tumor-like non-neoplastic lesions, with different cell types, can thus involve the skull base [1]. Skull base lesions that present with sinonasal extension are diagnostically challenging due to their anatomical complexity and nonspecific clinical features. Diagnosis cannot be confirmed on simple imaging in most cases [2]. While invasive aspergillosis is classically associated with immunocompromised hosts, it has also been rarely reported in immunocompetent individuals, particularly in endemic regions. Such presentations are uncommon and can closely mimic neoplastic processes, thereby posing significant diagnostic challenges. The pathogenesis involves inhalation of fungal spores, which, under favorable conditions, such as impaired mucociliary clearance, local tissue hypoxia, or immunosuppression, can proliferate and invade vascular and bony structures.

Clinically, invasive aspergillosis may present with nonspecific symptoms such as nasal obstruction, headache, proptosis, facial pain, or cranial neuropathies, depending on the site of extension. These features overlap considerably with sinonasal neoplasms, particularly in young patients, creating significant diagnostic ambiguity. Radiologically, both fungal granulomas and tumors may appear as enhancing soft tissue masses with bony erosion, further complicating differentiation. Tumors such as juvenile nasopharyngeal angiofibroma, chordoma, and inflammatory myofibroblastic tumor can resemble invasive fungal infections like chronic granulomatous fungal sinusitis on imaging, especially when bone erosion and intracranial or orbital extension are present [3].

This case details a 19-year-old male with a skull base-extending lesion initially diagnosed as a spindle cell neoplasm on biopsy, later found to harbor invasive fungal elements upon repeat sampling. It underscores the importance of adequate tissue sampling, early fungal cultures, special stains, and multidisciplinary interpretation to avoid misdiagnosis in complex skull base pathology.

## Case presentation

A 19-year-old male presented with a one-year history of gradually progressive swelling in the left periorbital region, accompanied by the recent onset of bilateral visual blurring and nasal obstruction. There was no history of immunodeficiency, diabetes mellitus, or steroid use. The patient also had no prior history of prolonged antibiotic use, recurrent sinusitis, or systemic immunosuppression. He denied fever and chronic headache. Family history was non-contributory, and there was no occupational exposure to organic or agricultural dust. Thus, the presentation was atypical, occurring in an otherwise healthy, immunocompetent young adult. Neurological examination revealed mild bilateral proptosis, more prominent on the left, without cranial nerve palsy.

**Figure 1:**
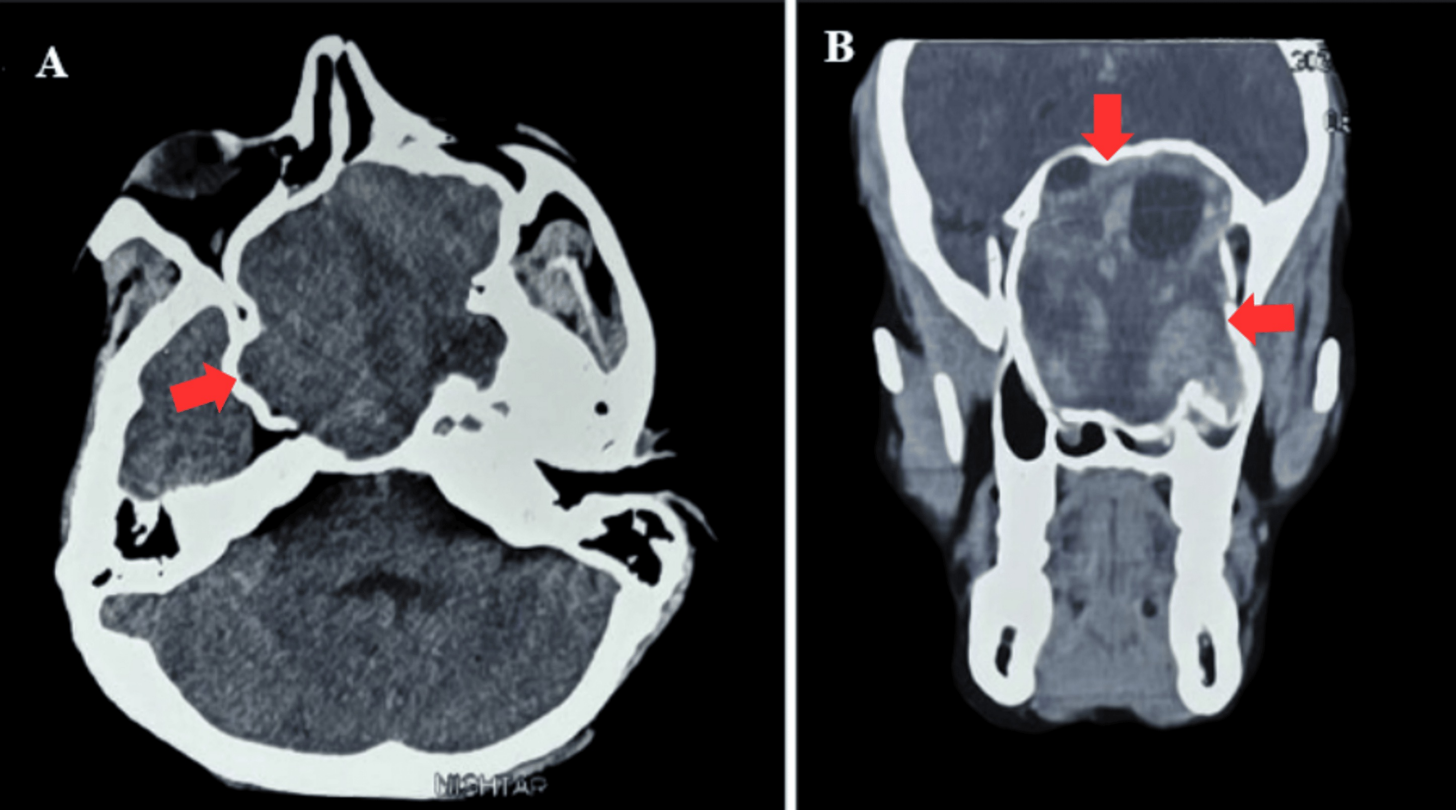
Axial CT image (A) showing skull base mass (red arrow) compressing the adjacent structures. Coronal CT image (B) showing proptosis caused by a mass involving bilateral nasal cavities and orbital floors (red arrows).

**Figure 2:**
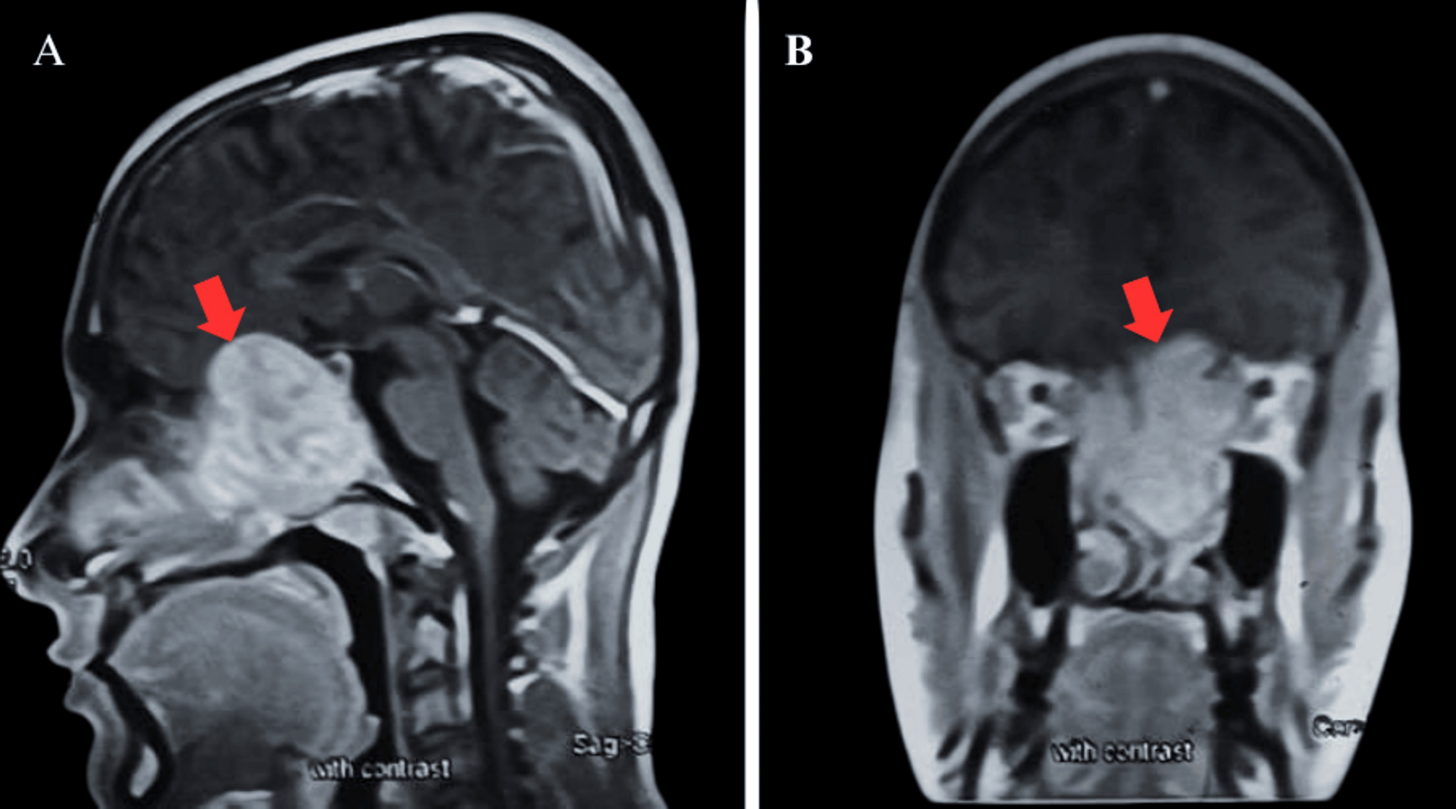
Coronal (A) and sagittal (B) MRI with contrast showing enhancing lesion (red arrows) compressing bilateral optic nerves and reaching orbital apices.

Contrast-enhanced CT (Figure 1) and MRI (Figure 2) of the brain revealed a large, lobulated, heterogeneously enhancing soft-tissue mass (71 x 68 mm) centered at the skull base involving the clivus, bilateral sphenoethmoidal sinuses, left maxillary sinus, and nasopharynx. The lesion extended anteriorly into the bilateral nasal cavities and superiorly up to the superior orbital fissures, causing narrowing and compressive effects on both optic nerves. CT angiography showed the lesion was vascularized by the cavernous segments of bilateral internal carotid arteries (ICAs). The primary radiologic differentials included juvenile nasopharyngeal angiofibroma and fungal granuloma.

**Figure 3:**
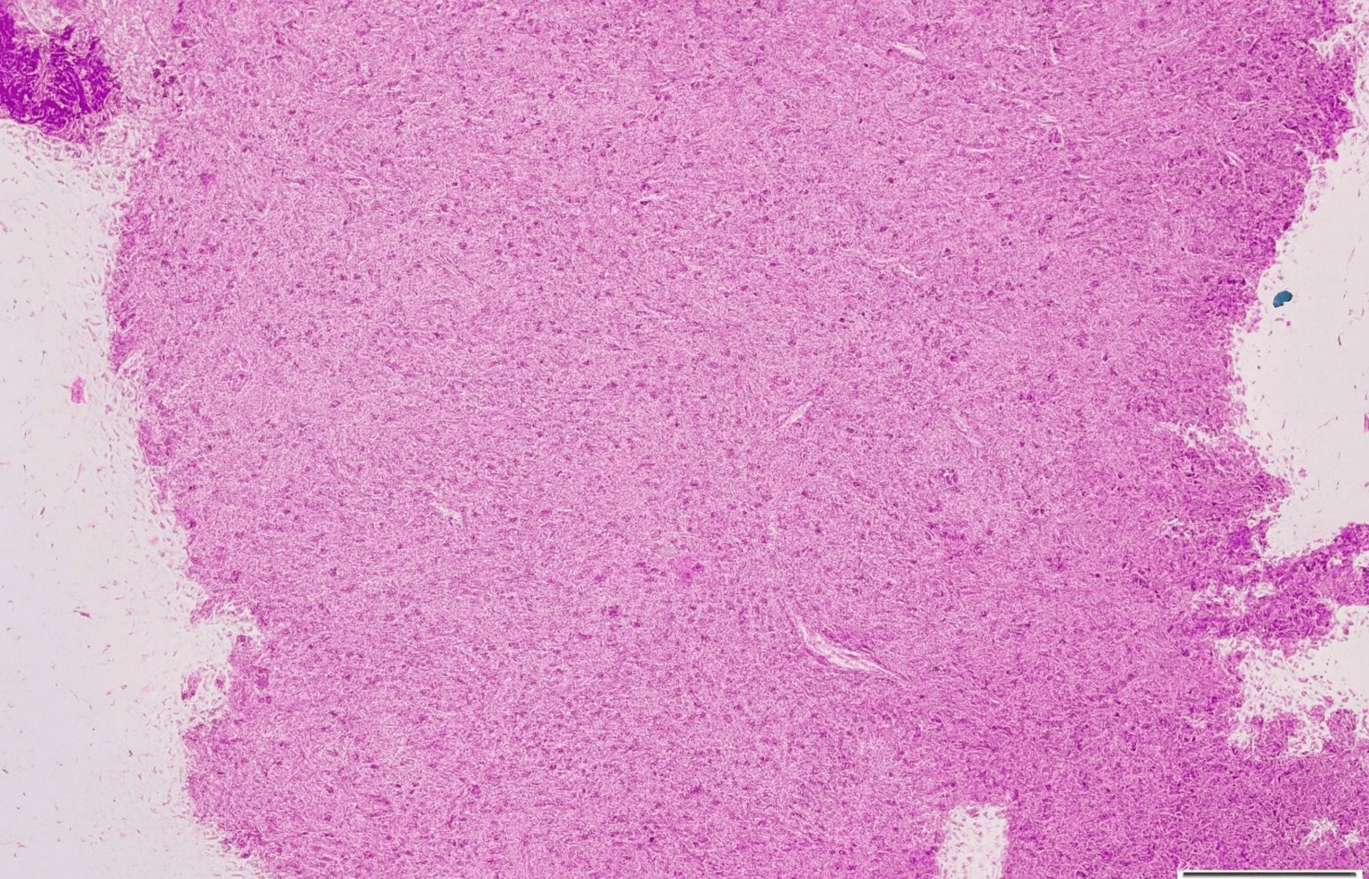
Histological examination of section reveals multiple fragments showing proliferation of stellate to spindle shaped cells having bland nuclear morphology.

An endonasal biopsy was performed under general anesthesia on August 29, 2024. Histopathological examination revealed spindle cell proliferation within a collagenous stroma (Figure 3), suggestive of a spindle cell neoplasm. Immunohistochemistry was performed using EMA, S100, SMA, and CD34 to evaluate for meningioma, schwannoma, inflammatory myofibroblastic tumor, and vascular lesions, respectively; however, staining was non-contributory and failed to establish a definitive diagnosis. The report recommended a repeat, larger biopsy to definitively exclude a juvenile nasopharyngeal angiofibroma. Concurrent tissue was submitted for fungal culture, which showed no growth.

The patient was lost to follow-up but returned four months later with worsening left proptosis and progressive visual deterioration. Repeat imaging revealed increased lesion size. A second endonasal biopsy was performed. Histopathology revealed necrotizing granulomatous inflammation with abundant fungal hyphae consistent with invasive fungal sinusitis. Gomori methenamine silver (GMS) and periodic acid-Schiff (PAS) stains confirmed septate, acute-angle branching hyphae consistent with *Aspergillus* species. 

The patient was started on voriconazole and underwent surgical excision. Postoperative follow-up showed gradual resolution of proptosis and visual symptoms.

## Discussion

*Aspergillus fumigatus* is a common environmental fungus that exhibits several biological characteristics, making it highly successful as a pathogen. It uses specialized surface molecules to attach to host tissues, and its cell wall structure is constantly remodeled, allowing it to avoid immune detection and adapt to the host’s internal environment [4]. While invasive aspergillosis usually affects immunocompromised individuals, there have been several documented cases in immunocompetent people. The precise mechanism by which the fungus invades in these cases remains uncertain. Instances of sino-orbital and sinonasal aspergillosis in immunocompetent patients have been reported, emphasizing the unclear nature of the disease's development in such individuals [5,6].

Fungal granuloma mimicking tumors are diagnostically challenging, particularly when involving the skull base, where their radiologic appearance and tissue invasiveness may suggest malignancy [7]. These fungal pseudotumors, although well-described in the context of mycobacterial infections, are relatively rare for fungal pathogens such as *Aspergillus*. At the histological level, the spindle cell morphology seen in biopsy specimens may represent reactive fibroblastic proliferation or an inflammatory pseudotumor rather than true neoplasia [8].

Similar diagnostic dilemmas have been reported in the literature. In one study, skull base aspergillomas were initially misdiagnosed as chordomas and meningiomas due to their radiological resemblance and fibrous histology [9]. Another case series has reported that invasive sphenoid sinus aspergillosis can closely mimic vascular or neoplastic lesions due to overlap in imaging features such as bony erosion, hyperdensity, and heterogeneous enhancement [7]. Clinical features such as facial swelling, proptosis, and visual involvement, as reported in our case, are also reported in tumors like juvenile nasopharyngeal angiofibroma [10]. CT angiography in our case showed the lesion to be vascularized by the ICA, which is also a common finding in juvenile nasopharyngeal angiofibroma [11]. These findings reinforce the importance of correlating radiological appearances with clinical and histopathological data, particularly in non-immunocompromised patients or those in endemic regions.

In our patient, the first biopsy revealed a spindle cell lesion without any fungal elements. It was only on repeat biopsy, prompted by progression of symptoms, that fungal hyphae were identified with PAS and GMS stains. *Aspergillus fumigatus* typically grows rapidly within two to three days on Sabouraud dextrose agar, producing blue-green to gray colonies with a powdery surface and a characteristic musty odor. This highlights that a single, small, or superficial sample may fail to capture the full spectrum of the pathology.

Early and accurate diagnosis is critical, as invasive fungal infections can rapidly cause significant morbidity due to optic nerve involvement, vascular encasement, or cavernous sinus extension. In such cases, prompt surgical debridement and antifungal therapy such as voriconazole have been shown to improve prognosis [12]. Our patient responded well to this approach, with resolution of the lesion on imaging and symptomatic improvement at follow-up.

## Conclusions

Skull base lesions can be deceptive, often imitating more familiar conditions. While juvenile nasopharyngeal angiofibroma is a common first consideration in young males with vascular skull base masses, clinicians must also remain alert to the possibility of invasive fungal granuloma. Its ability to mimic neoplastic disease, both radiologically and histopathologically, is well documented and should be considered regardless of immune status.

This case also highlights the limitations of early investigations. A superficial or limited biopsy may yield misleading results, as occurred here with the initial spindle cell impression. Nondiagnostic results should not be accepted as conclusive. When a lesion continues to progress, repeat biopsy with fungal stains and cultures is essential to establish the correct diagnosis.

Clinicians should maintain a high index of suspicion for fungal infections, even in immunocompetent individuals, particularly in endemic regions. Prompt recognition and combined surgical-medical management are critical to prevent irreversible complications.
